# Editorial: Advances in neuromodulation treatment of Parkinson's disease and aging-related movement disorders

**DOI:** 10.3389/fnagi.2024.1407216

**Published:** 2024-04-10

**Authors:** Kai-Liang Wang, Fangang Meng, Shinyuan Chen, Adolfo Ramirez-Zamora, Yu-Qing Zhang

**Affiliations:** ^1^Department of Neurosurgery, Xuanwu Hospital, Capital Medical University, Beijing, China; ^2^International Neuroscience Institute (China-INI), Clinical Research Center for Epilepsy, Capital Medical University, Beijing, China; ^3^China National Medical Center for Neurological Diseases, Beijing, China; ^4^Beijing Neurosurgical Institute, Capital Medical University, Beijing, China; ^5^Department of Neurosurgery, Hualien Tzu Chi Hospital, Buddhist Tzu Chi Medical Foundation, Hualien, Taiwan; ^6^Program in Movement Disorders and Neurorestoration, Department of Neurology, Fixel Center for Neurological Diseases, University of Florida, Gainesville, FL, United States; ^7^Department of Functional Neurosurgery, Xuanwu Hospital, Capital Medical University, Beijing, China

**Keywords:** Parkinson's disease, neuromodulation, deep brain stimulation (DBS), movement disorder, biomarkers

In recent years, significant strides have been made in the field of neuromodulation for the treatment of Parkinson's Disease (PD) and aging-related movement disorders. The intersection of neuroscience, technology, and medical innovation has paved the way for novel approaches to patients facing these debilitating conditions. From deep brain stimulation (DBS) to non-invasive neuromodulation techniques, the landscape of treatment options is rapidly evolving, promising improved symptom management and quality of life for individuals affected by these neurological disorders. This Research Topic “*Advances in neuromodulation treatment of Parkinson's disease and aging-related movement disorders*” explores the latest advances in neuromodulation therapies, related reviews summarizing, and assessment of disease progression, highlighting the potential prospect for managing aging-related movement disorders.

The topic is comprised of 12 articles including nine original articles and three review papers. These articles mainly focused on the effect of brain stimulation and physical activity on PD, the role of metabolites and receptors in PD, predictive factors, and the application of new approaches to PD progression.

The first reported randomized, double-blind, sham-controlled study to investigate the effectiveness of non-invasive vagus nerve stimulation (nVNS) in improving gait and other motor symptoms of PD patients was performed by Mondal et al.. Thirty-three PD patients experiencing freezing of gait (FOG) received either active nVNS or a placebo treatment. Remarkable enhancements in walking factors were observed with the use of active nVNS. Moreover, there were significant changes in serum tumor necrosis factor-α, glutathione, and brain-derived neurotrophic factor levels following the active nVNS treatment. The results suggested that nVNS could serve as a supplementary treatment in managing PD symptoms, particularly for FOG. Furthermore, Liu et al. performed a meta-analysis involving 16 randomized controlled studies with 408 patients to explore the impact of transcranial magnetic stimulation (TMS) on FOG in PD. The result showed that TMS was beneficial in enhancing gait and motor performance, while further researches are needed to explore the most effective stimulation parameters for TMS.

Except for applied stimulations, interactive interventions like action observation training (AOT) and physical activity, have proven to be effective in improving both cognitive abilities and motor skills for PD. Meng et al. conducted a study involving 30 early-stage PD patients, aiming to examine changes in brain functional connectivity (FC) and clinical outcomes after 12 weeks of Tai Chi-based action observation training (TC-AOT) in comparison to traditional physical therapy (TPT). Patients with TC-AOT displayed significantly higher FCs between specific brain regions. Furthermore, these changes in FCs were positively correlated with improvements in both motor and cognitive abilities. The result indicated that the TC-AOT enhanced the early-stage rehabilitation outcomes of PD by fostering brain neuroplasticity. Meanwhile, a study conducted by Lin et al. observed that the consumption of fish oil supplements and physical activity have both been linked to a lower risk of developing PD. Furthermore, the protective effect of physical activity against PD appears to be even stronger when combined with the use of fish oil supplements.

The study by Zeng et al. illustrated the progression of research focused on surgical interventions for tremors in PD patients between 2002 and 2022. DBS for PD tremor is still a research hotspot. Several concerns regarding DBS like operative indications, targets, and protocols associated should also be considered. Furthermore, magnetic resonance-guided focused ultrasound (MRgFUS) has become a hopeful treatment option for PD tremors. Consistent with Zeng et al. and Hong et al. also found that DBS was a reliable therapy for Young Onset Parkinson's Disease (YOPD). A total of 27 YOPD patients who underwent STN-DBS experienced obvious enhancements in their emotional wellbeing, with no negative impact on their cognitive abilities after a follow-up of 2 years. Honma et al. also investigated the contribution of the STN-DBS in temporal processing. The result suggested STN participated in the encoding of time duration and the role of time perception might be restricted to the externalization of memories acquired through experiences. STN-DBS may potentially enhance the functioning of the prefrontal cortex by modulating the basal ganglia-thalamo-cortical circuit.

Multiple research studies have found a connection between PD and reduced levels of uric acid (UA). Low UA levels have been linked to an increased risk of developing PD, as well as the progression and severity of the disease. Using resting-state functional MRI, Chang et al. investigated the connection between FC related to UA and outcomes of STN-DBS in PD patients. They found that PD patients with abnormal brain connections related to UA are strongly linked to the effectiveness of STN-DBS. Combining the two biomarkers of UA and FC provides neurosurgeons with valuable tools to identify the most suitable candidates and forecast the prognosis of PD patients. Another study by Shin et al. explored the effects of UA on the transfer of extracellular α-synuclein (α-syn) between cells and the protection of dopaminergic neurons in a model enriched with α-syn. The result indicated that UA could potentially manage the progression of PD by targeting multiple pathways that regulate the spread of α-synuclein. Yu et al. summarized the development of the cannabinoid type-2 receptor (CB_2_ receptor). The CB_2_ receptor was reported to have a potential impact on iron transport, oxidative stress, neuroinflammation, and neuronal cell death, which may play a role in the treatment of PD.

Early detection and early treatment of PD symptoms are essential. New approaches are emerging to play a role in predicting the advancement of PD. Hu et al. clarified the connection between the development of impulse control disorder (ICD) and the advancement of PD. Patients with different patterns of ICD evolution had varying changes in white matter microstructure at the onset of PD. The brain regions affected by these changes are known to play a role in both ICD and non-motor Functions. These patterns may also serve as predictive markers for the progression of motor symptoms and cognitive decline in PD patients. Visuospatial and cognitive dysfunction are prevalent among PD patients, which was also proven by the study of Shao et al.. The assessments conducted through the application (APP) demonstrated increased sensitivity and specificity, aiding clinicians in the swift and accurate diagnosis of PD patients with visuospatial disorders. The result indicated the potential of APP to provide early rehabilitation strategies and pharmacological interventions.

In conclusion, with the rapid development of neuromodulation, innovative findings continue to emerge. There is great hope for promising treatments for PD and aging-related movement disorders.

## Author contributions

K-LW: Writing – original draft, Writing – review & editing. FM: Conceptualization, Project administration, Resources, Supervision, Writing – review & editing. SC: Project administration, Resources, Supervision, Writing – review & editing. AR-Z: Resources, Supervision, Writing – review & editing. Y-QZ: Project administration, Resources, Supervision, Validation, Writing – original draft, Writing – review & editing.

